# Inflammation-Mediated Lipid Metabolism in Endocrine Autoimmune Diseases: A Genetic Distance-Based PRS Approach Integrating HLA Region

**DOI:** 10.3390/genes16111379

**Published:** 2025-11-12

**Authors:** Fenghuixue Liu, Yifei Ren, Wenhua Liu, Qi Chen, Ping Yin, Peng Wang

**Affiliations:** 1 School of Public Health, Tongji Medical College, Huazhong University of Science and Technology, Wuhan 430030, China; m202375720@hust.edu.cn (F.L.); m202475873@hust.edu.cn (Y.R.);; 2 Clinical Research Center, Tongji Hospital, Tongji Medical College, Huazhong University of Science and Technology, Wuhan 430030, China

**Keywords:** polygenic risk score, lipid metabolite, autoimmunity, inflammation, genetic epidemiology

## Abstract

**Background**: Endocrine autoimmune diseases (AIDs) exhibit special polygenic characteristics in human leucocyte antigen (HLA) region. Current understanding of their association with lipid metabolism remains constrained by imprecise polygenic risk score (PRS) modeling. Advanced analytical approaches are needed to elucidate the association between genetic susceptibility and lipid metabolic dysregulation. **Methods**: We proposed a genetic distance-based clumping gPRS to account for linkage disequilibrium in the HLA region. gPRS and pathway gPRS were constructed for individuals diagnosed with type I diabetes (T1D), Graves’ disease (GD), Hashimoto thyroiditis (HT) and Addison’s disease (AD) in the UK Biobank, with sex considered as a stratification factor. Latent correlations between gPRS and phenotypes were explored using Kendall’s tau test, two-trait LD score regression (LDSC) and gene annotation. **Results**: Lipid metabolism served an important function through immune and inflammatory biomarkers across multiple traits. Males with low genetic risk tended to have lower high-density lipoprotein cholesterol level, while the correlation presented the opposite pattern in females. Increased genetic susceptibility to AIDs was associated with elevated levels of low-density lipoprotein cholesterol, triglycerides in low-density lipoprotein (LDL) and very-low-density lipoprotein (VLDL) across all traits. Moreover, levels of polyunsaturated fatty acids, including omega-3 and omega-6, decreased with higher PRS in males and females, while those of monounsaturated fatty acids exhibited an increasing trend. **Conclusion**: Our study constructed more precise polygenic risk scores of AIDs, highlighting inflammation-mediated lipid metabolism as a potential pathogenic mechanism in endocrine AIDs, offering valuable insights into shared etiology for future comprehensive investigations.

## 1. Introduction

Autoimmune diseases (AIDs) are often accompanied by various uncharacteristic syndromes as a consequence of poly-autoimmunity [1]. Among these, lipid metabolic disorders are prevalent and predispose patients to a spectrum of associated complications. In the pancreas, adults with type I diabetes (T1D) exhibit an elevated risk for atherosclerotic cardiovascular diseases [2]. In the content of the thyroid, hypercholesterolemia often occurs in patients with moderate to severe Graves’ disease (GD) [3], while dyslipidemia often presents along with the hypofunction of thyroid [4] in the case of Hashimoto thyroiditis (HT). Approximately 85% of Addison’s disease (AD) cases are attributed to autoimmunity, and patients may suffer from several vague symptoms due to glucocorticoid deficiency in adrenal gland [5].

This commonality might contribute to inflammation—a fundamental mechanism in immune response—as lipids play a crucial role in the inflammatory process. Systematic lipotoxicity can induce inflammation via stress kinases and signaling proteins, and reciprocally, the release of cytokines and decrease in anti-inflammatory adipokines can alter the immune phenotype of adipose tissue, leading to exacerbated recruitment of immune effector [6]. Studies have demonstrated that lipids can modulate adaptive immunity by influencing T cell function and fate [7], and are used as fuel to support anti-tumor immune responses [8]. However, studies directly addressing the involvement of these mechanisms in autoimmune diseases are relatively sparse. Although the lipid profile of macrophages at the onset of T1D has been observed in mouse models [9], the role of inflammation-mediated lipid metabolism in humans remains to be fully elucidated.

Genetic epidemiology has provided new approaches for association studies to explore the latent mechanism connecting genotype, endophenotype and phenotype [10]. Polygenic risk score (PRS) provides a quantitative genetic evaluation for individuals by summarizing weighted allele scores in an additive model [11]. However, the construction of PRS faces challenges in AIDs, as they are heavily influenced by the human leukocyte antigen (HLA) region [12], which is in high linkage disequilibrium (LD), and this strong correlation structure across the genome introduces bias into the estimation of independent effects. Traditional methods use a physical distance of 250 kb as a clumping window to extract independent and significant single-nucleotide polymorphisms (SNPs) [13]. This is limiting for the 8000 kb HLA region [14], since the use of such a narrow window can lead to loss of crucial information within the HLA region. Unfortunately, this issue has often been overlooked for autoimmune diseases. As the simple expansion of window size could lead to poor statistical power, some studies simply excluded the HLA region [15], while others avoid SNP selection and adjust effect sizes globally, utilizing methods such as PRS-CS [16]. To fully leverage original genome-wide association studies (GWAS) and address these limitations, our study introduces a novel approach that uses genetic distance, rather than physical distance [17] for clumping to preserve the integrity of the HLA region. This study will construct PRS models that account for the genetic architecture of endocrine AIDs to elucidate the role of immune and inflammatory biomarkers in lipid metabolism. By focusing on the multi-systemic and poly-glandular characteristics of endocrine AIDs, our study can further explore the common genetic architecture and metabolic patterns across traits, providing a deeper understanding of the underlying pathology.

## 2. Materials and Methods

### 2.1. Study Population, Genetic and Phenotype Data

This study utilized the demographic, disease diagnosis, genotype and blood sample data from the UK Biobank (Project 162275), a large-scale biobank which contains an extensive array of variables collected from 502,143 participants aged 40 to 69 years. We constrained ethnicity to British and Irish, and ascertained disease diagnosis based on ICD-10 main diagnosis (T1D: E10; GD: E05.0; HT: E06.3; AD: E27.1). Individuals with sex discrepancies and genotype heterozygosity values exceeding 3 standard deviations from the mean were excluded. The control group was derived from a population without AIDs or a similar phenotype. Summary data from genome-wide association studies (GWAS) were obtained from the GWAS Catalog, as detailed in Supplementary Table S1. Plasma lipid metabolites encompass lipoprotein components of different densities, as well as the ratio of diverse fatty acids to total fatty acids. Immune and inflammatory biomarkers include IL6, IL9, IL17A, CD40, TGFβ, HLA-A, HLA-E, and HLA-DRA.

### 2.2. Data Quality Control and Imputation

Quality control procedures for individual genetic data were implemented as described. Genotyping was performed using the Applied Biosystems UK BiLEVE Axiom Array by Affymetrix (Santa Clara, CA, USA). Imputation was carried out using the R package snp_fastImputeSimple in R 4.3.2. SNP filtering was conducted with PLINK 1.9 software, which removed SNPs that met any of the following criteria: genotyping call below 98%, missing rate exceeding 0.1, minor allele frequency (MAF) less than 1%, deviation from Hardy–Weinberg equilibrium (HWE, *p* < 0.001), or a coefficient of kinship greater than 0.125. Only autosomal chromosomes were included. Quality control indices are listed in Supplementary Table S2.

Quality control of GWAS summary data followed the established tutorial guideline [18]. SNPs were harmonized with the HAPMAP3+ reference panel, and those with ambiguous base pairs, duplicate IDs, MAF below 0.01, or extreme sample sizes and chi-squared values were excluded.

### 2.3. Genetic Distance-Based Clumping for PRS

To comprehensively consider the HLA region in the clumping procedure, we proposed a clumping and threshold selection method based on genetic distance (gPRS). Physical distances (kb) were initially converted to genetic distances (cM) according to the genetic map released by the Genome Reference Consortium Human GRCh38. We employed a 3 cM clumping window for LD matrix computation to fully capture the HLA region and maintain computational efficiency, a strategy shown to be robust by Privé et al. [17]. To choose the optimal selection threshold among 1 × 10^−8^, 5 × 10^−8^, 1 × 10^−5^, 5 × 10^−5^, 0.001, 0.01, 0.05, 0.1, 0.2, and 0.5, we randomly allocated participants into training and test datasets at a ratio of 8:2, and calculated the area under the curve (AUC) of each model considering sex and phenotype. PRS adjusted for the top 20 genetic principal components was ultimately incorporated.

To demonstrate the utility of our gPRS approach, we replicated the analysis, excluding the HLA region, and compared it with traditional clumping methods, as well as PRS-CS.

### 2.4. Statistical Analysis

We employed the K-Nearest Neighbors (KNN) imputation method to address missing data. The gPRS was stratified into five discrete categories for each sex, defined as follows: bottom 10% (<10th percentile), low 30% ([10th, 40th) percentile), middle 20% ([40th, 60th) percentile), high 30% ([60th, 90th) percentile), and top 10% (≥90th percentile). Correlations between the categorized gPRS and each lipid metabolite were assessed utilizing Kendall’s tau test.

Sensitive analyses were conducted to explore the mediation effect of immune and inflammatory biomarkers. We constructed pathway gPRS based on SNPs within the genetic regions associated with immune and inflammatory biomarkers, as annotated in dbSNP, and repeated the aforementioned examination between the pathway gPRS and lipid metabolites. LDSC [19] was also conducted to explore the genetic correlation between diseases and lipid metabolites on a genome-wide scale. GWAS data for LDSC are provided in Supplementary Table S1. Statistical analyses were conducted in R 4.4.1 and Python 3.8.

### 2.5. Ethics Approval and Consent to Participate

UK Biobank was approved as a tissue bank resource by the North West Multi-centre Research Ethics Committee. All UK Biobank participants gave written informed consent for use of their data for health research. Participants who withdrew consent during this study were excluded from the analysis.

## 3. Results

### 3.1. Baseline Characteristics of Participants

A total of 4245 participants with T1D, 907 with GD, 377 with HT, 349 with AD and 36,093 controls were enrolled in the study. Females exhibited a higher prevalence among cases. Age, lipid metabolites, as well as immune and inflammatory biomarkers were found to be similar between cases and controls, as detailed in Table 1 and Supplementary Table S3.

### 3.2. Distribution of Polygenic Risk Score and Model Performance

The AUCs of the performed models are presented in Table 2. The gPRS model demonstrated robust performance across T1D, GD, HT and AD. The inclusion of the HLA region yielded higher AUCs, suggesting better fitness. gPRS outperformed conventional clumping methods in T1D, GD, and HT, and exhibited superior performance to PRS-CS in T1D and HT. In GD and AD, the AUCs were slightly lower but still comparable. Given the enhanced predictive accuracy of the gPRS model incorporating the HLA region, subsequent analyses were conducted based on it.

**Figure 1:**
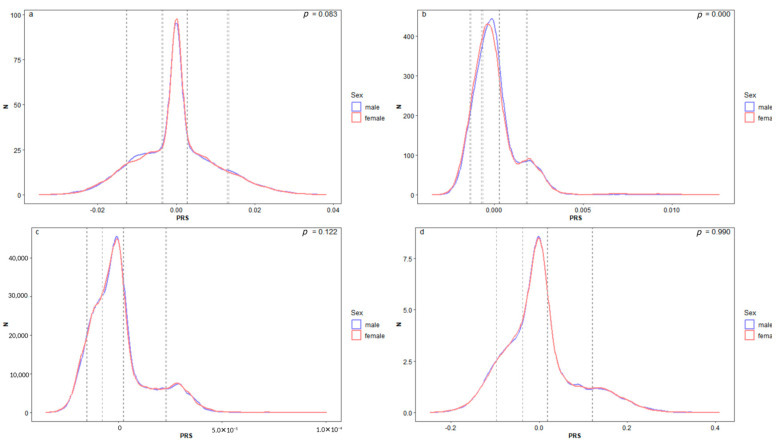
Sex stratified distribution of PRS. It presents the gPRS distribution for two sexes across T1D (**a**), GD (**b**), HT (**c**), and AD (**d**). The solid blue and red lines delineate the distribution for males and females, respectively. The dashed lines indicate the boundaries for the top 10%, the high 20%, the middle 20%, the low 30%, and the bottom 10% within each sex. The *p*-value of Kolmogorov–Smirnov test between sexes is shown in the top right.

The PRS distribution for each disease is shown in Figure 1. The PRS distributions for both sexes did not exhibit significant differences in T1D, HT, and AD. For GD, the median PRS was higher (*p* < 0.001) in males (median = −1.77 × 10^−4^) than in females (median = −2.47 × 10^−4^).

### 3.3. Common Genetic Architecture Across Traits

**Figure 2:**
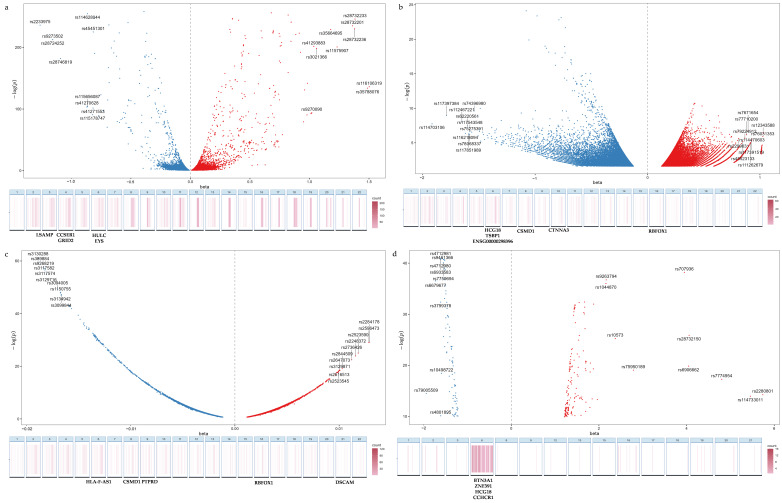
Distribution of fitted SNPs illustrates the distribution of SNPs and gene enrichment annotation associated with T1D (**a**), GD (**b**), HT (**c**), and AD (**d**). In the volcano plots, the X-axis represents the effect size of each SNP, while the Y-axis denotes the significance level as indicated by −log(*p*) values. SNPs are color-coded to reflect their effect direction: positive (red) and negative (blue), with further distinction between those located within the HLA region and those outside of it, as detailed in the legend. The heat maps below are organized by chromosome and depict the concentration of SNPs within genes. The top five most enriched gene regions are explicitly annotated.

The effect sizes and significance of the fitted SNPs are depicted in Figure 2a–d. In the case of AD, a substantial 31% of SNPs were located within the HLA region. A similar pattern was also observed, albeit to a lesser extent, in T1D with 30%, HT with 23%, and GD with 20%. These findings underscore the pivotal role of the HLA region in the genetic architecture of these diseases. We also located the SNPs into functional gene regions referring to the UCSC database, and the top five enriched genes for each disease were detailed in Figure 2a–d. Within the HLA region, the strongest association signal for AD was at the BTN3A1 locus, while for HT it was at HLA-F-AS1. For loci outside of the HLA region, HCG18 gene on chromosome 6 was commonly implicated in both GD and AD, whereas in the case of GD and HT variants were highly enriched in CSMD1 on chromosome 8.

### 3.4. Polygenic Risk Score and Blood Lipid Metabolites

**Figure 3:**
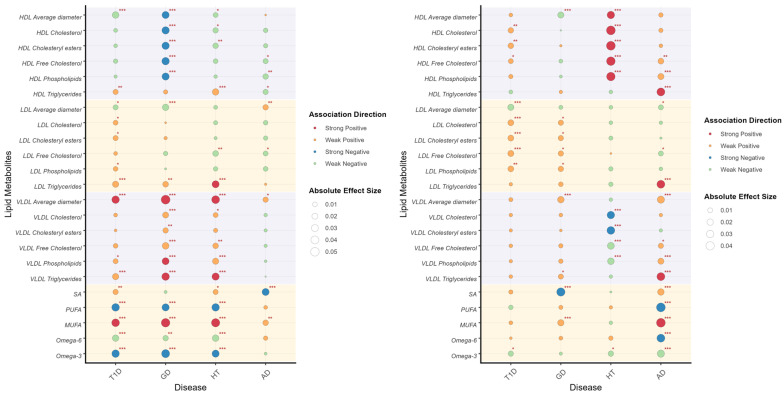
Correlation between PRS and lipid metabolites illustrates the correlation between PRS and lipid metabolites in males (**left**) and females (**right**). Lipid metabolites are categorized through different background color. The association direction of PRS and lipid metabolites are indicated by colors, as shown in the legend. The absolute effect sizes are visualized by the diameter of circles. Significant values are specially addressed by corner marks. *: *p* < 0.05; **: *p* < 0.01; ***: *p* < 0.001.

We assessed the relationship between genetic risk and lipid metabolite levels across T1D, GD, HT, and AD to identify metabolites that were either shared or specific to individual diseases (Figure 3). The majority of these associations exhibited the same direction in both sexes, though the magnitude and significance varied.

HDL particles demonstrated significant associations with the PRS of multiple endocrine AIDs. Cholesterol components, including cholesteryl esters, free cholesterol, and phospholipids, exhibited strong negative correlations with GD risk in males and positive correlations with HT in females. Notably, triglycerides within LDL emerged as an independent risk factor for T1D, GD, and HT in males, and AD in females. Among multiple traits, the strongest correlation association was observed between LDL components and female T1D PRS, highlighting sex-specific disease mechanisms. VLDL particles were strongly associated with genetic risk in male GD and HT, suggesting shared metabolic pathways. Moreover, VLDL triglycerides demonstrated significant associations across all four diseases and in both sexes. Sex-specific patterns were prominent in fatty acid-PRS relationships. In males, a higher polyunsaturated fatty acid (PUFA) ratio (omega-6/omega-3) was inversely correlated with autoimmune risk, whereas saturated (SFA) and monounsaturated fatty acids (MUFA) showed positive correlations. The estimate, 95% confidence intervals, and *p* values are listed in Supplementary Table S4.

### 3.5. Mediation Effect of Immune and Inflammatory Biomarkers

**Figure 4:**
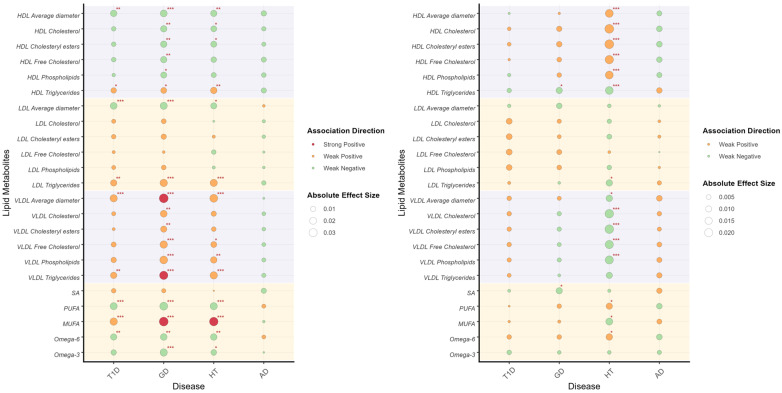
Correlation between pathway PRS and lipid metabolites illustrates the correlation between pathway PRS and lipid metabolites in males (**left**) and females (**right**). Lipid metabolites are categorized through different background color. The association direction of pathway PRS and lipid metabolites are indicated by colors, as shown in the legend. The absolute effect sizes are visualized by the diameter of circles. Significant values are specially addressed by corner marks. *: *p* < 0.05; **: *p* < 0.01; ***: *p* < 0.001.

We constructed pathway PRS restricted to the HLA-region SNPs associated with inflammatory mediators, and evaluated their correlations with lipid metabolites (Figure 4). These analyses corroborated the previously observed association patterns, explaining 62.4% of the correlation in T1D (95%CI = 0.475, 0.773) and a lesser extent in AD (beta = 0.022, 95%CI = −2.500, 2.544) and GD (beta = 0.081, 95%CI = −1.839, 1.677). However, the result of HT (beta = 1.091, 95%CI = 0.791, 1.391) indicated the possible existence of counterfactual framework. Specifically, the associations between HDL cholesterol components and male GD PRS, as well as female HT PRS were predominantly mediated by inflammatory pathways. The inverse relationships observed for LDL and VLDL triglycerides likely reflect inflammatory modulation of lipid metabolism. Notably, significant associations for fatty acids were almost exclusively maintained in males, being largely absent in females.

### 3.6. Two Traits LDSC

Genetic correlations were calculated on the GWAS scale using LDSC (Table 3). AD was excluded due to the unavailability of high-quality GWAS. The findings generally align with those observed in individual-scale studies. Genetic correlations were conservative in T1D and GD, in contrast to the analysis of HT, which yielded significant results.

## 4. Discussion

Our study proposed a gPRS approach that effectively accounts for LD in the HLA region, demonstrating superior performance in modeling endocrine AIDs. Applying this method, we delineated the relationships between gPRS, lipid metabolites, and immune-inflammatory pathways, thereby identifying shared genetic and metabolic architectures across T1D, GD, HT, and AD.

Given the long-range regulatory mechanism of SNPs and complex LD patterns, models based solely on fixed physical windows can be insufficient in capturing extended genomic relationships [20]. Our proposed gPRS method converts physical distance to genetic distance in the clumping procedure, which notably improved the performance of PRS models. This is particularly applicable to polygenic diseases with high contribution of the HLA region such as AIDs. Moreover, AIDs exhibit strong heritability; for instance, the heritability of T1D is estimated between 72% and 88% in European populations [21]. This high genetic predictability makes our approach particularly well suited for these conditions. Recent evidence not only indicates shared pathophysiological mechanisms in AIDs but has also identified relevant biomarkers, such as lipid metabolites and chronic inflammation factors [22], highlighting the need to explore their common genetic architecture with advanced polygenic approaches. However, gPRS also exhibited skewness from the standard Gaussian distribution in GD and HT, which may stem from moderate quality of GWAS data, limited sample size of individual data, underlying strong LD, and the assumption of an additive genetic model.

Our study located several shared highly enriched genetic loci, most of which have been proven to play an inflammatory role in AIDs. Koyama et al. [23] found that CCHCR1 is significantly correlated with alopecia areata, a tissue-specific AID. In line with this finding, the identification of CCHCR1 in our analysis further supports its broader role in autoimmunity. BTN3A1 is widely implicated in AIDs [24]. It upgrades the expression of leukocytes [25] in innate immunity and contributes to adaptive immunity by activating specific T cells via phosphoantigen [26]. These immune responses are accompanied by differential gene expression related to lipid metabolism [27], revealing the inseparable function of lipids in AIDs. HLA-F-AS1, a long non-coding RNA (lnRNA), is found to be upregulated in polycystic ovary syndrome [28]. It is identified to increase the proliferation of granulosa cells, which acts through glucose and lipid metabolites and subsequently induces a range of endocrine symptoms [29]. Similarly, the overexpression of another lnRNA, PTPRD, induces inflammation through the upregulation of inflammation biomarkers such as TGFβ and IL1β [30], which play a role in the process of cholesterol loading [31]. Conversely, some of the identified regions exhibit a protective effect. Overexpression of HULC can decrease the levels of IL1, IL6 and IL8 [32], mitigate dyslipidemia [33], and alleviate atherosclerosis [34]. The upregulation of GRID2 can inhibit the invasion of tumor-associated immune cells [35]. Specifically, several loci in the HLA region were also identified, emphasizing the necessity of integrating the HLA region. CSMD1 is identified as being related to brain and neurological diseases [36] in previous GWAS. It acts on the metabolism of branched chain amino acids, fatty acids and cholesterol by regulating methylmalonate levels [37]. A recent study also revealed that obstructing the expression of CSMD1 can effectively suppress the secretion of inflammation factors including IL-6 and IL-8 [38]. HCG18 is normally identified as a tumor differentiation regulating gene [39]. Surprisingly, it also presents significant relatedness with total cholesterol in patients with non-alcoholic fatty liver disease [40], which might explain its role in AIDs. Our pathway PRS, including HLA-A, HLA-E, and HLA-DRA, reaffirms the importance of the HLA region, which is corroborated by previous studies in T1D [41], GD [42], and HT [43]. Generally, our findings highlight several loci of endocrine AIDs and indicate the underlying pathology of immune and inflammatory pathways. These shared features can account for their multi-systemic and poly-glandular characteristics and broaden the scope of future investigation. However, it is important to acknowledge that our annotation method may have limitations, since over 90% of GWAS trait-associated SNPs fall in non-coding regions [44]. Utilizing eQTL and fine mapping [45] could potentially refine and enhance our findings.

Beyond gene localization, to further bridge these genetic findings to their functional relevance, we investigated how the constructed gPRS correlates with quantitative measures of lipid metabolism. HDL is known for its role in reverse cholesterol transport, which involves esterifying free cholesterol to form mature HDL particles. This process exerts anti-oxidative and anti-inflammatory effects by modulating cytokines [46]. Our study found that males with low genetic risk tend to have lower HDL cholesterol components, while the correlation presented the opposite in females, revealing different immunological responses induced by same SNPs across sexes. This sex-specific trend in HDL level was also observed by Yang [47] et al. One possible explanation is that some genes such as HLA DR3 are regulated by sex specifically [48]. Moreover, spatial transcriptome analysis also indicated that females exhibit higher levels of inherent plasma B cells in autoimmune thyroid models compared to males [49], thus leading to sexually dimorphic lipoprotein functionality.

VLDL transports esterified triglycerides to peripheral tissues and acts as a precursor to LDL, with its remnants directly contributing to low-grade inflammation [50] by activating pro-inflammatory pathways such as NF-κB [51]. Genetic correlation between lipidemic traits and psoriasis, a prevalent AID [52], was also previously observed, which enhances our hypotheses of the association between lipid components and AIDs. This pathway might be explained by the inflammatory cascade brought by the elevation of triglycerides within LDL and VLDL particles [50], leading to autoimmune responses and metabolic syndrome. Notably, endogenous estrogens mitigate triglyceride and cholesterol abnormalities in pre-menopausal women, yet this protective effect diminishes post-menopause [53]. As our study focuses on an elderly cohort, the interference of endogenous estrogens is effectively minimized. This allows us to reasonably ascribe the observed sex differences to genetic impact.

PUFAs, particularly omega-3 fatty acids, give rise to pro-inflammatory lipid signaling molecules, which may be related to the upregulation of MHC I [54]. These two metabolites consistently decrease across all traits, reinforcing that the risk alleles of endocrine AIDs can promote the formation of inflammatory environment. MUFAs are believed to have anti-inflammatory properties [55] and are supposed to decrease with inflammation risk, which is inconsistent with our results. However, recent evidence suggests that they can exacerbate inflammation in specific contexts [56], which might explain the elevated MUFA levels observed across all diseases in our research. The function of MUFA in auto-immunology remains to be fully discovered.

Overall, we proposed a genetic distance-based clumping method (gPRS) to calculate PRS, which outperformed traditional methods for endocrine AIDs. Additionally, our correlation analyses were conducted across four traits, T1D, GD, HT, and AD, which provide mechanistic anchors for exploring endocrine autoimmunity as an interconnected disease spectrum. In summary, our work generates testable hypotheses regarding shared pathways in endocrine AIDs pathogenesis and might inform future investigations into biomarkers and therapeutic targets.

There are also several limitations that warrant consideration. Firstly, our model construction is not yet capable of fully addressing the impact of LD, which may introduce collinearity into our model and cause false-positive results. Secondly, our findings were derived from a European-ancestry cohort and may have limited applicability to other populations due to differences in linkage disequilibrium and allele frequencies. Thirdly, our analysis relied on baseline assessments from the biobank to maximize data completeness, future research could incorporate longitudinal data and a broader range of confounding variables, including pleiotropy, SNPs on sex chromosomes and other environmental factors to account for the complex interplay influencing autoimmune diseases. Finally, our genetic exploration based on PRS was relatively rough, and the statistical associations lack functional validation. More sophisticated methods in omics research and improved validation frameworks can be employed to elucidate the common genetic architecture underlying endocrine AIDs.

## Figures and Tables

**Table 1:** Demographic characteristics of AIDs.

	T1D	GD	HT	AD	Control
age	58.586 (7.671)	56.323 (7.971)	56.906 (7.825)	57.442 (8.032)	56.363 (8.049)
sex					
males	2475 (58.3%)	180 (19.9%)	29 (7.6%)	141 (40.4%)	16,959 (46.9%)
females	1770 (41.6%)	727 (80.1%)	348 (92.3%)	208 (59.5%)	19,134 (53.0%)
total	4245	907	377	349	36,093

**Table 2:** Area under the curve of each model.

	gPRS	gPRS_nHLA	C + T	PRS-CS
T1D	0.724	0.717	0.578	0.618
GD	0.758	0.709	0.687	0.766
HT	0.856	0.801	0.741	0.805
AD	0.564	0.555	0.608	0.583

gPRS and gPRS_nHLA indicate gene distance-based clumping method accounting and excluding the HLA region, respectively. C + T refers to traditional clumping method with threshold selection.

**Table 3:** LDSC results for diseases and lipid metabolisms.

	T1D		GD		HT	
	Genetic Correlation	*p*	Genetic Correlation	*p*	Genetic Correlation	*p*
HDL Cholesterol	−0.065 (0.050)	0.195	−0.055 (0.038)	0.155	−0.091 (0.038)	0.017
Cholesteryl esters in HDL	0.110 (0.120)	0.360	0.123 (0.091)	0.175	0.056 (0.093)	0.552
Free cholesterol in HDL	−0.132 (0.067)	0.048	−0.032 (0.056)	0.571	−0.252 (0.064)	<0.001
Cholesteryl esters in VLDL	0.030 (0.062)	0.630	0.125 (0.052)	0.017	0.265 (0.059)	<0.001
Average Diameter of VLDL	−0.039 (0.064)	0.542	0.052 (0.061)	0.396	0.203 (0.062)	0.001
Omega3 Fatty Acids	−0.075 (0.048)	0.113	−0.028 (0.043)	0.518	0.029 (0.039)	0.456
PUFAs	−0.023 (0.067)	0.728	−0.078 (0.062)	0.208	−0.285 (0.061)	<0.001
MUFAs	0.024 (0.064)	0.703	0.073 (0.059)	0.218	0.306 (0.061)	<0.001

## Data Availability

Data from the UK Biobank Resource were accessed under Application Number 162275. GWAS data is available from: https://www.ebi.ac.uk/gwas/docs/api (accessed on 6 December 2024). HAPMAP3 data is available from: https://www.broadinstitute.org/medical-and-population-genetics/hapmap-3 (accessed on 30 October 2024). The original contributions presented in this study are included in the article/Supplementary Materials. Further inquiries can be directed to the corresponding authors.

## Supplementary Materials

The following supporting information can be downloaded at: https://www.mdpi.com/article/10.3390/genes16111379/s1, Table S1: GWAS information; Table S2: Information of Target data after QC; Table S3: Baseline Characteristic; Table S4: Kendall test of PRS and lipid metabolites; Table S5: Kendall Test of pathway PRS and lipid metabolites.

## Abbreviations

The following abbreviations are used in this manuscript:

T1D	Type1 diabetes
GD	Graves’ disease
AD	Addison’s disease
HT	Hashimoto’s thyroiditis
PRS	Polygenic risk score
HLA	Human leucocyte antigen
LD	Linkage disequilibrium
SNPs	Single-nucleotide polymorphisms
GWAS	Genome-wide association studies
MAF	Minor allele frequency
HWE	Hardy–Weinberg equilibrium
gPRS	Genetic distance-based clumping polygenic risk score
GLM	Generalized linear model
AUC	Area under the curve
KNN	K-nearest neighbors
HDL	High-density lipoprotein
VLDL	Very low-density lipoprotein
PUFAs	Polyunsaturated fatty acids
MUFAs	Monounsaturated fatty acids
lnRNA	Long non-coding RNA
